# Burnout, satisfaction and happiness among German general practitioners (GPs): A cross-sectional survey on health resources and stressors

**DOI:** 10.1371/journal.pone.0253447

**Published:** 2021-06-18

**Authors:** Lena Werdecker, Tobias Esch

**Affiliations:** Institute for Integrative Health Care and Health Promotion, Faculty of Health, School of Medicine, Witten/Herdecke University, Witten, Germany; Faculty of Health Sciences - Universidade da Beira Interior, PORTUGAL

## Abstract

Well-being is a major issue among health care professionals, especially physicians. Less job satisfaction and impaired health can have an impact on health care quality. Our aim was to examine the association of stressors (illegitimate tasks) and health related resources (work-related sense of coherence; recovery experience) with life satisfaction, happiness, job satisfaction and burnout among German general practitioners (GPs). We conducted a cross-sectional survey among general medical practices in Germany. Main outcome measures were life satisfaction, happiness (Subjective Happiness Scale), job satisfaction (Work Satisfaction Scale) and burnout (Copenhagen Burnout Inventory). 548 GPs from across Germany participated (53.6% males, 45.6% females; mostly representative of German GPs). One third (35.2%) of the participants reported a high prevalence of personal, and one quarter (26.5%) indicated a high prevalence of work-related burnout symptoms. Illegitimate tasks are negatively associated with life and job satisfaction and are positively associated with personal, work-related and patient-related burnout among GPs. Work-SoC and recovery experience are positively associated with life satisfaction, happiness, and job satisfaction and are negatively associated with personal, work-related and patient-related burnout. Female physicians have a higher job satisfaction than male physicians. Being female and working as an employed physician is associated with a higher prevalence of personal burnout symptoms. GPs working in a group practice are happier and more satisfied with their job than GPs in single practices. Personal, work-related and patient-related burnout symptoms are stronger in GPs working in a single practice than in GPs in group practices. Our results highlight that Work-SoC, recovery experience and illegitimate tasks are important for creating work-related well-being among GPs. Introducing health promotion activities which aim to strengthen recovery experience and Work-SoC, as well as interventions to restructure tasks, may increase life satisfaction, happiness, and job satisfaction and reduce burnout symptoms in this health care profession.

## Introduction

In recent decades, especially during the COVID-19-pandemic, work-related stress, mental health issues and general well-being among physicians and other health care professionals have shifted to the top of the agenda [1–5]. The term “unhappy doctors” [6] was coined in the early 2000s [7, 8]. Subsequent studies pointed out that not only the individuals themselves are affected by decreased wellness, but that the quality of health care delivery may be affected as well, e.g. due to errors or inefficacy [9–12].

In many countries, GPs and their teams are the primary contact point for the general population. A growing number of elderly patients with chronic diseases and multimorbidity, patients with psychosocial needs and an imminent shortage of GPs in Germany contribute to an increase in workload in general care practice [13, 14]. Administrative tasks are an additional aspect related to well-being and job satisfaction among GPs [14, 15]. Trends in primary care, e.g. the tendency to work as an employee and preference for working in cities rather than in rural areas [16], call for monitoring the well-being of GPs.

Therefore, we conducted a study aming to explore the well-being among GPs in Germany. We examined life satisfaction and happiness as general traits and job satisfaction as a positive experience and an evaluation of different aspects of one’s job [17]. As burnout is a major concern among physicians [18], we included this phenomenon additionally. In this study, burnout is mainly understood as a psychological phenomenon resulting from continuous exposure to work-related stress. According to the stress theory of allostatic load by McEwen (2017, [19]), chronic stress can have pathophysiological consequences. We are interested in fatigue and exhaustion related to work, in particular to work with patients, but also in general as defined by Kristensen and colleagues [20]. Other burnout symptoms include cynicism, depersonalization and perceived inefficacy [21]. Burnout is associated with several psychological and physical health conditions such as cardiovascular disorders and depression [22]. A prominent model to explain the underlining mechanism for developing burnout in the work context is the job demands-resources theory [23]. This theory assumes that job demands such as emotional, social and physical demanding situations together with high workload are risk factors for exhaustion. However, this model also proposed that job resources such as control at work and personal resources such as self-efficacy could moderate the impact of job demands on burnout.

Still, many studies on mental health at work focus on the pathogenic paradigm. Less attention has been paid to health-related resources in research on well-being among GPs and health care professionals in general [24]. Health-related resources such as optimism, reflective skills, flexibility and self-efficacy, attitudes, or social support can help to manage stressors in general care practice [24–26]. Knowledge of health-related factors important for well-being among GPs may be used to fuel the development of interventions designed a) to increase life satisfaction, happiness, and job satisfaction and decrease burnout among GPs, b) to enhance retention among GPs in order to stabilize the primary health care setting (in Germany), and c) to make general care practice more attractive for future physician generations. This study therefore aims to display the association of stressors (illegitimate tasks) and health-related resources (work-related sense of coherence (Work-SoC); recovery experience) with life and job satisfaction, happiness and burnout among German GPs.

Research on job satisfaction among GPs often refers to high levels of administration and documentation effort [14, 27]. These administrative tasks can be described as “illegitimate tasks” [28]. Illegitimate tasks (unreasonable and unnecessary tasks) are stressors and potential risk factors for burnout and lower job satisfaction among GPs [28–31]. Illegitimacy of tasks is associated with higher stress perception, e.g. due to threats to self-esteem, or a subjective loss of control. Hence, such tasks are negatively associated with health-related resources like control or social support, and they can reduce the meaningfulness of one’s job. Previous studies have shown that illegitimate tasks are associated with emotional exhaustion and sickness presenteeism [29, 32, 33].

We also included Work-SoC and recovery experience as resources in our study, as they are potentially able to promote well-being. Work-SoC describes a situation in which one’s work is experienced as meaningful, comprehensive and manageable [34]. It is supposed that a higher Work-SoC can help to better understand work-related stressors and is facilitating to successfully handle them [35, 36]. Likewise, recovery experiences may be important facilitators for unwinding from job stressors; they include strategies of psychological detachment, relaxation, mastery experience, and control during leisure time [37]. A high Work-SoC and high recovery experience after work are expected to be positively associated with job satisfaction [34] and general well-being [37, 38], but negatively associated with burnout. Sonnentag/Lischetzke [39] examined the association of illegitimate tasks and recovery experience and concluded that “illegitimate tasks are associated with unfavorable states at the end of the workday and are indirectly related to poor psychological detachment from work, undermining recovery from the stressful events experienced at work”.

The main research questions of this study are

- How satisfied are GPs with their lives and their job? How happy are they? How prevalent is burnout among GPs?
- How are illegitimate tasks, Work-SoC and recovery experience associated with life satisfaction, happiness, job satisfaction and burnout among GPs whilst controlling for variances in gender, age, type of practice, and employment status?

## Methods

Primary care in Germany is typically organized by independent private practices in which one or more physicians (practice owner/self-employed, employed) work together (single or group practice, less in medical centers). GPs are organized at federal level in an association of statutory health insurance physicians. In 2020, 55,012 physicians worked as GPs in Germany [13].

### Study population

In our data analyses, we included 548 physicians (GPs, internal medicine), practicing in general medicine/primary care. We excluded other primary care physicians such as pediatricians, gynecologists and other specialists, as these specialties are not considered primary care in some countries. 10,000 GPs were randomly chosen by an independent partner (ArztData AG) along the first two digits of the zip code (01xxx, 02xxx, …) to draw a stratified sample across Germany.

### Data collection

We invited GPs and their teams (colleagues and staff) by mail to participate in our cross-sectional survey study. Participants had the choice between using an online link (LimeSurvey) provided in the invitation letter and sending a fax to order paper-pencil surveys. 302 GPs (55.1%) used the paper-pencil survey, 246 GPs (44.9%) participated in the online survey. The data was collected from August to November 2018. Given the fact of reaching the calculated sample size of 372 GPs (α = 0.05 and β = 0.20; anticipated response rate of 5%) after the first reminder, we stopped the recruitment.

### Ethical considerations

Ethical approval was given by the ethical review committee of Witten/Herdecke University (No. 134/2017). The data was collected in line with the Declaration of Helsinki. Participation was voluntary and the survey was anonymous. Participations who answered the online survey had to confirm their consent before answering the questions. Participants completing the paper-pencil survey consented by returning the anonymous questionnaire.

### Instruments

Life satisfaction was measured by one item “All in all, how satisfied are you currently with your life?” [40]. This question is also included in the household based longitudinal German socio-economic panel. We used a 10-point Likert scale ranging from 0 “not at all satisfied” to 9 “completely satisfied”.

Happiness was measured by the German version of the Subjective Happiness Scale [41, 42]. The scale consisted of four items. Two items asked participants to rate themselves generally from 1 “not a very happy person” to 7 “a very happy person” and in comparison with peers from 1 “less happy” to 7 “happier”. The other two items used a short characterization of happy and unhappy people and participants were asked to indicate to what extent the description applied to themselves (from 1 “not at all” to 7 “a great deal”). The Subjective Happiness Scale has already been used among health care professionals [43, 44]. We calculated the mean of all four items after reverse coding of the fourth item. A higher score equates to a higher level of happiness.

Job satisfaction was measured by the German version of the job satisfaction scale [45] developed by Warr, Cook, and Wall [46], modified by Cooper et al. for GPs [47]. In nine items, participants were asked to rate different work parameters (such as physical work load, colleagues, remuneration) on a 7-point Likert scale ranging from 1 “very unsatisfied” to 7 “very satisfied”, while one item asked for an overall rating of job satisfaction. The scale has already been used in studies among GPs and other health care workers [48–51]. We calculated the mean of all ten items. A higher score equates to a higher level of job satisfaction.

Burnout was measured by the Copenhagen Burnout Inventory (CBI) [20]. A core strength of this scale is that it differentiates between three levels of exhaustion: personal (6 items), work-related (7 items) and client-related (6 items). The CBI shows a high correlation with the Maslach Burnout Inventory (MBI) subscale of exhaustion [2, 52] and has been used in multiple studies among health care professionals [53–59]. We applied the German version [52] by changing the wording of the subscale “client-related” to “patient-related“. Participants rated their response on a 5-point Likert scale with two different dimensions (intensity and frequency). We calculated the mean for each subscale. To compare the published findings with our results we also dichotomized the burnout variable according to Borritz and colleagues [60] in the middle of the scale (low prevalence of burnout symptoms < 50; high prevalence of burnout symptoms ≥ 50).

Illegitimate tasks were measured by the Berne Illegitimate Task Scale (BITS) [28, 29, 61, 62]. Performing illegitimate tasks, i.e. tasks that are not considered core tasks, is a stressor for a professional’s role identification. Thun and colleagues [33] applied the BITS to physicians in a hospital setting. The scale is divided into two subscales: unnecessary (4 items) and unreasonable tasks (4 items). We calculated the mean for each subscale and the mean of the sumscore. A higher score equates to a higher frequency of these tasks during the usual workload.

Work-related Sense of Coherence (Work-SoC) was measured by the German version of the Work-related Sense of Coherence Scale [34] which consists of 9 items and 3 subscales: manageability (2 items), meaningfulness (3 items) and comprehensibility (4 items). Perception is ranked by assessing contrary adjectives on a 7-point bipolar Likert scale. We calculated the mean for each subscale and the sumscore. A higher score equates to a higher Work-SoC.

The ability to recover after work is measured by the Recovery Experience Questionnaire (REQ). Recovery experience is an indicator of how individuals are able to distance themselves from work-related stressors. The scale is divided into four subscales [37]: psychological detachment from work (4 items), relaxation (4 items), mastery (4 items) and control (4 items). The perception of each item after work is measured on a 5-point Likert scale. We calculated the mean for each subscale and the sumscore. A higher score equates to a stronger ability to recover.

Furthermore, we asked for details relating to the participants’ profession, age, gender, type of practice, place of practice, employment status, volume of work, and time in outpatient care. The total survey included further instruments. However, these results are presented elsewhere.

### Handling of missing values

In this study, we only included data of instruments for which less than 5% of data were missing. Missing data are indicated in descriptive statistics. Correlation and regression analyses were performed listwise. The total number of cases included is reported for each analysis. For comparisons, we performed the regression analyses after multiple imputation (n = 20) in SPSS (Fully Conditional Specification Method). To calculate imputations we included all variables which also fed into the three models. F-statistics and adjusted R^2^ were calculated as means of all 20 imputations. The results are reported in the S1 and S2 Tables.

### Analyses

We used SPSS Statistics 26 for the data analyses. To demonstrate the internal reliability of the instruments, we calculated Cronbach’s alpha for the totals and subscales [63]. We calculated absolute and relative frequencies for nominal data. Rating scales with at least five points we interpreted as interval data [64], so that we calculated means and standard deviation. Bivariate correlations were performed using Pearson’s correlations (listwise). The effect size was rated according to Cohen [65]. P-values of <0.05 were considered statistically significant. Multiple, linear regression models were calculated to assess the association of independent variables with burnout. We checked the variance inflation factor (VIF) to detect collinearity [66] and we report beta, 95% confidence intervals and standardized betas, adjusted R^2^ and F-statistics for each model.

## Results

We stopped disseminating the questionnaire after reaching the calculated sample size; in the end, a total of 548 physicians participated in our study. The study population consisted of 53.6% males and 45.6% females. The mean age of physicians was 54.38 years (SD 9.2 years). Most survey participants worked in single practices, were self-employed and worked full time (Table 1). The detailed results of the scales are presented in the S3 Table.

**Table 1:** Description of the study population (n = 548).

		N	%				
Gender	Female	250	45.6				
	Male	294	53.6	Female (n = 250)		Male (n = 294)	
	*Missing data*	4	0.7	N	%	N	%
**Age group**	20 to 29 years	1	0.2	0	0	1	0.3
	30 to 39 years	32	5.8	25	10.0	7	2.4
	40 to 49 years	110	20.1	61	24.4	49	16.7
	50 to 59 years	250	45.6	122	48.8	128	43.5
	60 to 69 years	127	23.2	41	16.4	86	29.3
	70 to 79 years	22	4	1	0.4	21	7.1
	Older than 80 years	2	0.4	0	0.0	2	0.7
	*Missing data*	4	0.7	0	0.0	0	0.0
**Type of practice**	Single practice	274	50	120	48.0	154	52.4
	Group practice	245	44.7	114	45.6	128	43.5
	Medical centre	24	4.4	13	5.2	11	3.7
	*Missing data*	5	0.9	3	1.2	1	0.3
**Place of practice**	Rural municipality	194	35.4	80	32.0	113	38.4
	Small town	176	32.1	76	30.4	99	33.7
	Medium-sized town	74	13.5	38	15.2	35	11.9
	Metropolis	97	17.7	52	20.8	45	15.3
	*Missing data*	7	1.3	4	1.6	2	0.7
**Employment status**	Self-employed	472	86.1	193	77.2	279	94.9
	Employed	70	12.8	52	20.8	15	5.1
	*Missing data*	6	1.1	5	2.0	0	0.0
**Volume of work**	Full time	461	84.1	182	72.8	278	94.6
	Part time	80	14.6	66	26.4	12	4.1
	*Missing data*	7	1.3	2	0.8	4	1.4
**Time in outpatient practice**	Less than 10 years	114	20.8	74	29.6	39	13.3
	10 to 20 years	184	33.6	84	33.6	99	33.7
	20 to 30 years	156	28.5	74	29.6	81	27.6
	More than 30 years	90	16.4	15	6.0	75	25.5
	*Missing data*	4	0.7	3	1.2	0	0.0

As highlighted in Table 2 below, the bivariate correlations indicate a negative correlation between illegitimate tasks and life satisfaction, happiness, and job satisfaction on the one hand. Furthermore, Table 2 indicates positive correlations between the three burnout levels and illegitimate tasks. On the other hand, it reveals positive correlations between life satisfaction, happiness, and job satisfaction and Work-SoC and recovery experience. Negative correlations are shown between the three burnout levels and Work-SoC and recovery experience.

**Table 2:** Descriptive statistics and correlations (Pearson coefficients, listwise, n = 478) for study variables.

	*n*	*M*	*SD*	1	2	3	4	5	6	7	8	9
1. Life satisfaction^a^	544	6.80	1.81	** **								
2. Happiness^b^	546	5.14	1.13	0.468	0.69							
3. Job satisfaction^c^	530	5.32	0.99	0.511	0.405	0.87						
4. Personal burnout^d^	544	42.49	20.00	-0.464	-0.446	-0.535	0.88					
5. Work-related burnout^d^	541	37.07	19.19	-0.496	-0.488	-0.668	0.813	0.88				
6. Patient-related burnout^d^	541	26.62	18.17	-0.364	-0.362	-0.546	0.458	0.643	0.83			
7. Illegitimate tasks^e^	541	3.06	0.78	-0.317	-0.209	-0.528	0.423	0.534	0.426	0.91		
8. Work-SoC^f^	535	5.27	0.96	0.439	0.326	0.700	-0.534	-0.651	-0.494	-0.546	0.85	
9. Recovery experience^g^	529	3.33	0.65	0.337	0.390	0.371	-0.503	-0.481	-0.237	-0.154	0.310	0.91

All correlation coefficients are statistically significant at p< 0.01. M = Mean, SD = Standard deviation, Cronbach’s alpha is displayed on the diagonal.

^a^ Ranged from 0 “totally disagree” to 9 “totally agree”

^b^ Ranged from 1 “not happy/less happy/not at all” to 7 “very happy/happier/totally”

^c^ Ranged from 1 “very unsatisfied” to 7 “very satisfied”

^d^ Ranged from 100 “always or to a very high degree” to 0 “never/almost never or to a very low degree”

^e^ Ranged from 1 “never” to 5 “frequently”

^f^ Ranged from 1 “manageable/meaningless/structured/easy to influence/insignificant/clear/controllable/unrewarding/predictable” to 7 “unmanageable/meaningful/unstructured/impossible to influence/significant/unclear/uncontrollable/rewarding/unpredictable”

^g^ Ranged from 1 “totally disagree” to 5 “totally agree”.

**Fig 1:**
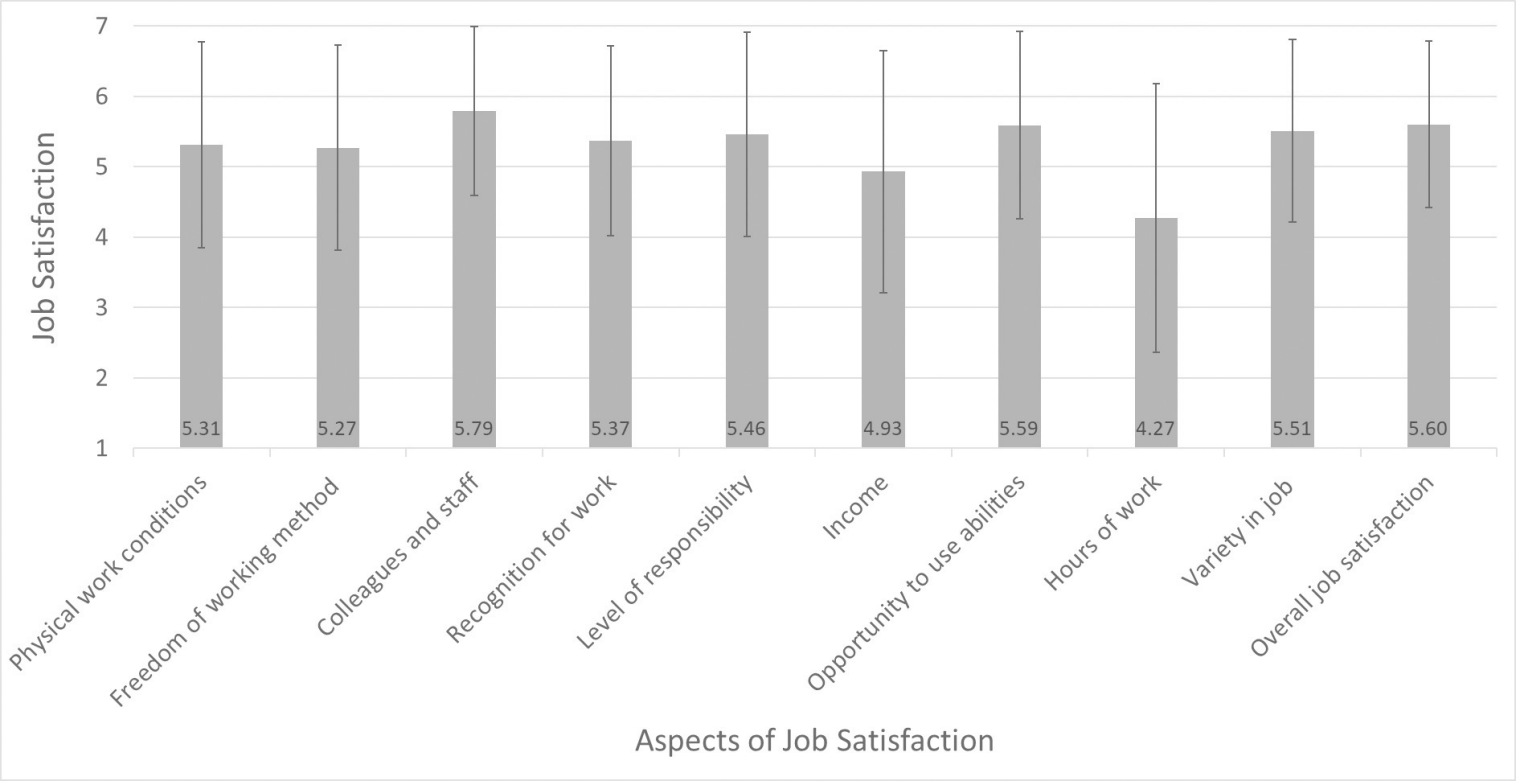
Aspects of job satisfaction among GPs. Means and standard deviations are shown for aspects of job satisfaction ranging from 1 “very unsatisfied” to 7 “very satisfied”.

GPs scored rather high for the overall job satisfaction (M = 5.60; SD = 1.18) as the average score for the prompted aspects is 5.28. Fig 1 illustrates the rating of job satisfaction aspects (results reported in the S4 Table). Interestingly, GPs are on average most satisfied with colleagues and staff (M = 5.79; SD = 1.20), the opportunity to use their skills (M = 5.59; SD = 1.33), and the variety of job-related tasks (M = 5.51; SD = 1.30). They are less satisfied with work hours (M = 4.27; SD = 1.91) and income (M = 4.93; SD = 1.72). It seems that the latter aspects matters less for their overall rating.

If we dichotomize the burnout score (< 50 and ≥ 50), we see that approximately one third (35.2%) of the participants reported a high prevalence of personal burnout symptoms, while one quarter (26.5%) indicated a high prevalence of work-related burnout symptoms. Almost 12% of the respondents reported a high prevalence of patient-related burnout symptoms (Table 3).

**Table 3:** Prevalence of burnout symptoms among GPs (n = 548).

		N	%
**Personal burnout**	Low prevalence of symptoms (< 50)	351	64.1
	High prevalence of symptoms (≥ 50)	193	35.2
	*Missing data*	4	0.7
**Work-related burnout**	Low prevalence of symptoms (< 50)	396	72.3
	High prevalence of symptoms (≥ 50)	145	26.5
	*Missing data*	7	1.3
**Patient-related burnout**	Low prevalence of symptoms (< 50)	476	86.9
	High prevalence of symptoms (≥ 50)	65	11.9
	*Missing data*	7	1.3

### Regression analyses

We performed multiple linear regression analyses with life satisfaction, happiness and job satisfaction (Table 4), with personal burnout, work-related burnout and patient-related burnout as dependent variables (Table 5), and illegitimate tasks, Work-SoC and recovery experience as independent variables. For the analyses, we controlled for gender, age, type of practice and employment status.

**Table 4:** Linear model of predictors of life satisfaction, happiness and job satisfaction among GPs.

	Life satisfaction (n = 496)						Happiness (n = 498)						Job satisfaction (n = 484)					
Effect	b	*SE*	β	95% CI		*p*	b	*SE*	β	95% CI		*p*	b	*SE*	β	95% CI		*p*
				*LL*	*UL*					*LL*	*UL*					*LL*	*UL*	
Intercept	2.778	0.886		1.037	4.518	**0.002**	2.081	0.570		0.962	3.201	**<0.001**	2.143	0.385		1.386	2.900	**<0.001**
Illegitimate tasks	-0.265	0.111	-0.114	-0.483	-0.047	**0.017**	-0.067	0.071	-0.046	-0.206	0.072	0.346	-0.234	0.048	-0.187	-0.329	-0.139	**<0.001**
Work-SoC	0.550	0.093	0.290	0.367	0.733	**<0.001**	0.239	0.060	0.201	0.122	0.356	**<0.001**	0.537	0.040	0.528	0.457	0.616	**<0.001**
Recovery experience	0.670	0.119	0.238	0.436	0.905	**<0.001**	0.540	0.077	0.305	0.390	0.691	**<0.001**	0.219	0.053	0.143	0.115	0.322	**<0.001**
Male^a^	-0.168	0.150	-0.046	-0.463	0.127	0.265	-0.102	0.096	-0.045	-0.291	0.087	0.290	-0.131	0.066	-0.067	-0.260	-0.003	**0.046**
Age	-0.004	0.008	-0.023	-0.021	0.012	0.603	0.003	0.005	0.025	-0.008	0.014	0.577	0.006	0.004	0.057	-0.001	0.013	0.105
Group practice^b^	0.178	0.147	0.049	-0.111	0.467	0.228	0.218	0.094	0.096	0.033	0.403	**0.021**	0.177	0.064	0.090	0.051	0.303	**0.006**
Medical care center^c^	0.181	0.373	0.021	-0.551	0.913	0.628	0.417	0.239	0.076	-0.053	0.887	0.082	0.097	0.161	0.021	-0.219	0.414	0.546
Employed^d^	-0.369	0.248	-0.067	-0.856	0.119	0.138	0.039	0.160	0.011	-0.275	0.354	0.806	-0.106	0.107	-0.036	-0.317	0.105	0.324

Bold = statistically significant at p< 0.05. b = unstandardized beta coefficient, SE = standard error of the unstandardized beta coefficient, β = standardized beta coefficient, p = p-value, CI = confidence interval, LL = lower level, UL = upper level.

^a^ 0 = Female, 1 = Male

^b^ 0 = Single practice, 1 = Group practice

^c^ 0 = Single practice, 1 = Medical care center

^d^ 0 = Self-employed, 1 = Employed. Life satisfaction: Adjusted R^2^ = 0.228 (F = 19.311, p< 0.001). Happiness: Adjusted R^2^ = 0.192 (F = 15.729, p< 0.001). Job satisfaction: Adjusted R^2^ = 0.509 (F = 63.506, p< 0.001).

**Table 5:** Linear model of predictors of burnout among GPs.

	Personal burnout (n = 496)						Work-related burnout (n = 495)						Patient-related burnout (n = 496)					
Effect	b	*SE*	β	95% CI		*p*	b	*SE*	β	95% CI		*p*	b	*SE*	β	95% CI		*p*
				*LL*	*UL*					*LL*	*UL*					*LL*	*UL*	
Intercept	102.242	7.965		86.593	117.892	**<0.001**	99.066	7.128		85.061	113.071	**<0.001**	59.688	8.606		42.777	76.598	**<0.001**
Illegitimate tasks	6.013	0.998	0.238	4.052	7.974	**<0.001**	6.419	0.895	0.261	4.661	8.177	**<0.001**	5.201	1.072	0.222	3.095	7.308	**<0.001**
Work-SoC	-5.680	0.836	-0.276	-7.322	-4.037	**<0.001**	-7.840	0.749	-0.392	-9.312	-6.368	**<0.001**	-6.399	0.904	-0.334	-8.174	-4.623	**<0.001**
Recovery experience	-11.106	1.083	-0.361	-13.234	-8.979	**<0.001**	-9.035	0.958	-0.305	-10.918	-7.152	**<0.001**	-2.989	1.155	-0.105	-5.260	-0.719	**0.010**
Male^a^	-4.215	1.352	-0.107	-6.871	-1.559	**0.002**	-0.072	1.208	-0.002	-2.446	2.302	0.952	1.750	1.454	0.048	-1.106	4.606	0.229
Age	-0.154	0.077	-0.072	-0.305	-0.002	**0.047**	-0.152	0.069	-0.073	-0.287	-0.017	**0.028**	-0.075	0.082	-0.038	-0.236	0.086	0.362
Group practice^b^	-3.659	1.323	-0.093	-6.258	-1.060	**0.006**	-3.877	1.180	-0.101	-6.196	-1.558	**0.001**	-4.641	1.422	-0.127	-7.435	-1.848	**0.001**
Medical care center^c^	-3.077	3.358	-0.032	-9.675	3.521	0.360	-2.698	3.029	-0.029	-8.650	3.254	0.374	-4.159	3.604	-0.047	-11.240	2.922	0.249
Employed^d^	6.796	2.228	0.114	2.418	11.174	**0.002**	0.006	1.992	0.000	-3.907	3.919	0.998	2.076	2.386	0.038	-2.613	6.764	0.385

Bold = statistically significant at p< 0.05. b = unstandardized beta coefficient, SE = standard error of the unstandardized beta coefficient, β = standardized beta coefficient, p = p-value, CI = confidence interval, LL = lower level, UL = upper level.

^a^ 0 = Female, 1 = Male

^b^ 0 = Single practice, 1 = Group practice

^c^ 0 = Single practice, 1 = Medical care center

^d^ 0 = Self-employed, 1 = Employed. Personal burnout: Adjusted R^2^ = 0.470 (F = 55.768, p< 0.001). Work-related burnout: Adjusted R^2^ = 0.554 (F = 77.664, p< 0.001). Patient-related burnout: Adjusted R^2^ = 0.294 (F = 26.789, p< 0.001).

Illegitimate tasks are negatively associated with life (β = -0.114, p = 0.017) and job satisfaction (β = -0.187, p< 0.001). Work-SoC and recovery experience are positively associated with life satisfaction, happiness, and job satisfaction. Female physicians have a higher job satisfaction than male physicians (b = -0.131, p = 0.046). No statistically significant associations were found between gender and life satisfaction or gender and happiness. The age variable did not show any statistically significant association with life satisfaction, happiness, or job satisfaction. GPs working in a group practice are happier (b = 0.218, p = 0.021) and more satisfied with their job than GPs working in single practices (b = 0.177, p = 0.006). The employed/self-employed variable did not show any statistically significant association with life satisfaction, happiness, and job satisfaction.

The model for explaining the variance in job satisfaction (adjusted R^2^ = 0.509; F = 63.506, p<0.001) shows the strongest fit.

After adjusting for gender, age, type of practice and employment status, illegitimate tasks are positively associated with personal (β = 0.238; p< 0.001), work-related (β = 0.261; p< 0.001) and patient-related burnout (β = 0.222; p< 0.001) among GPs. Work-SoC and recovery experience are negatively associated with personal (β = -0.276; p< 0.001; β = -0.361; p< 0.001), work-related (β = -0.392; p< 0.001; β = -0.305; p< 0.001) and patient-related burnout (β = -0.334; p< 0.001; β = -0.105; p = 0.010), whereas recovery experience shows the strongest association with all three levels of burnout.

Personal burnout symptoms are more prevalent in female than in male physicians (b = -4.215; p = 0.002). No statistically significant associations were found between gender and work-related or patient-related burnout. Age is negativley associated with personal (β = -0.072; p = 0.047) and work-related burnout (β = -0.073; p = 0.028). Personal (b = -3.659; p = 0.006), work-related (b = -3.877; p = 0.001) and patient-related burnout symptoms (b = -4.641; p = 0.001) are stronger in GPs working in a single practice than in GPs in group practices. No statistically significant association was observed for the medical center variable versus the single practice variable. Personal burnout symptoms are more prevalent in GPs working as employed physicians than in self-employed GPs (b = 6.796; p = 0.002).

The models for explaining the variance in personal (adjusted R^2^ = 0.470; F = 55.768, p< 0.001) and work-related burnout (adjusted R^2^ = 0.554; F = 77.664, p< 0.001) are almost equal in terms of model fit. However, the model for explaining the variance in patient-related burnout is weaker (adjusted R^2^ = 0.294; F = 26.789, p< 0.001).

## Discussion

To the best of our knowledge, this is the first study that examines the association of illegitimate tasks, Work-SoC and recovery experience with satisfaction, happiness and different levels of burnout among GPs. Illegitimate tasks are negatively associated with life and job satisfaction. Work-SoC and recovery experience are positively associated with life satisfaction, happiness, and job satisfaction. Recovery experience has a stronger conditional association with happiness, whereas Work-SoC is more strongly associated with life satisfaction and job satisfaction. Illegitimate tasks seem to increase burnout symptoms, while Work-SoC and recovery experience appear to reduce personal, work-related and patient-related burnout symptoms. Female physicians have a higher job satisfaction than male physicians. GPs working in a group practice are happier and more satisfied with their job than GPs in single practices. Being female and working as an employed physician is associated with a higher prevalence of personal burnout symptoms. As age increases personal and work-related burnout symptoms decrease. Personal, work-related and patient-related burnout symptoms are stronger in GPs working in a single practice than in GPs working in group practices.

The mean happiness score among German GPs was 5.14 (SD 1.13). In comparison with other studies using the SHS to measure happiness in physicians, German GPs seem to be happier than family physicians in the US [43, 44]. However, participants of a fellowship program for family physicians were happier than our study sample [43]. Previous studies that examined happiness among health care workers and students of health care professions report both lower but also higher levels of happiness [67–70]. Compared to findings of studies with other community samples, our happiness score ranks among the higher levels of happiness [42, 71, 72]. All in all, we can conclude that German GPs seem to belong to the happier proportion of the samples studied. We did not find any association between happiness and gender or age. This finding is in line with other studies which did not show any association with demographic variables either [41, 44, 71, 72]. However, a previous study among GPs showed that happiness is affected by gender [43].

Our finding regarding job satisfaction (mean of 5.32, SD = 0.99) is in line with other studies among GPs. Some previous studies report higher scores for job satisfaction [45, 73], other research measured lower scores [14, 48, 50, 51, 74]. Our results relating to satisfaction with different job aspects are in accordance with findings of other studies among GPs in Germany and other European health care systems [45, 48, 51, 73–75]. This study indicates that female GPs are happier than their male colleagues and that age is not associated with job satisfaction. Previous studies report inconsistent findings for gender and age differences [14, 48, 50, 73, 75, 76].

The fact that GPs report higher average scores in personal and work-related burnout symptoms than in patient-related burnout is in line with other studies [20, 52, 60, 77, 78]. Previous studies from Germany and other countries show that the scores for personal, work-related and patient-related burnout are lower among our study participants compared to other medical specialists, such as anesthetists or surgeons [54–57, 78, 79]. However, other studies report lower scores compared to our findings [20, 59]. Considering that an average score of over 50 is rated as strong burnout symptoms and that all average scores in our study are well below 50, we can conclude that burnout is not as severe among German GPs as among physicians in general [18]. Nevertheless, one third or one quarter of GPs with highly prevalent burnout symptoms are proportions which need to be further investigated, as they are indicators for health risks at work. In general, the findings relating to gender differences in burnout symptoms are varied and clear evidence is lacking [14, 80–83]. Studies using the CBI to measure burnout tend to find that women have a higher prevalence of burnout symptoms than men [78, 79, 84]. However, among these studies, some show gender differences in personal and work-related burnout but not in patient-related burnout [57, 77, 84]. The higher prevalence of personal burnout symptoms among female GPs may be explained by additional tasks, e.g. household and childrearing [81]. This assumption is supported by findings that indicate a poor work-life balance among female physicians [14, 85].The age variable showed statistically significant associations with personal and work-related burnout. The findings relating to associations between age and burnout symptoms are also inconsistent [2]. Some studies suggest that specific age groups, middle-aged and younger age groups, have a greater risk of developing burnout symptoms [14, 57, 83].

Our research highlights that GPs working in a group practice are happier and more satisfied with their job than GPs working in single practices. It corroborates a previous study [27] and our own findings regarding burnout. Personal, work-related and patient-related burnout symptoms were found to be more prevalent in GPs working in single practices than in GPs working in group practices. Previous studies that investigated differences between burnout symptoms and chronic stress among GPs in group or single practices are inconclusive [14, 80, 86, 87]. An explanation for our observation may be a poor work-life balance, higher workload and less frequent use of stress-regulating interventions such as exercise or socializing among GPs in single practices, or the lack of shared responsibilities in terms of billing, staff or patient care [14, 82]. Our results encourage further research on the correlation between work setting and personal wellness, including interactions in private life. Our finding that personal burnout is more prevalent among GPs working as employed physicians rather than as self-employed GPs is in line with results of another German study [82]. This observation may be explained by the assumption that employed physicians have less control over work-life interactions. Furthermore, it is possible that physicians deliberately choose to work as employee because they have other commitments, e.g. caring for family members. These commitments may contribute to higher levels of personal burnout symptoms among employed physicians working in group practices.

Overall, Work-SoC and recovery experience have not been examined among physicians thus far. However, the scores of Work-SoC in our study are consistent with findings of a study among Norwegian nursing home employees, even though they applied the Norwegian 8-item Work-SoC (5.34 vs. 5.27 in our study [88]). In addition, a German study with employees of different companies confirms our scores [34]. The score for recovery experience measured in our survey falls within the range of scores for other occupational groups [34, 89]. Our study adds to previous findings [34, 38, 88, 90] that Work-SoC and recovery experience positively affect life satisfaction, happiness, and job satisfaction among GPs. In contrast to our findings, a Norwegian study among nursing home employees found no association between Work-SoC and job satisfaction and assumed that Work-SoC contributes less to “passive states of well-being” [88].

Thun and colleagues [33] examined unreasonable tasks among Norwegian physicians and found similar scores as we did in our sample. However, they did not find a bivariate correlation between unreasonable tasks and exhaustion [33]. Our finding that GPs more often report unnecessary than unreasonable tasks is in line with other studies [31]. A comparison of our results with the findings of studies among other occupational groups suggests that GPs are more strongly affected by illegitimate tasks than other professions [17, 29, 31]. Our study adds evidence to previous findings that illegitimate tasks affect health outcomes [29, 32, 33].

### Implications for practice

In the context of occupational health and system-related aspects such as retention, job performance and quality of care, patient satisfaction, absenteeism and presenteeism, our findings are valuable for policymakers and the German Association of Statutory Health Insurance Physicians as these key players are responsible for ensuring access to primary care in their region/federal state.

Our findings highlight that we should develop different interventions for enhancing general and work-related well-being. Interestingly, happiness is associated solely by health-related resources and the type of practice. Our results suggest that health-promoting interventions focusing on Work-SoC and recovery experience may increase happiness in GPs to a certain extent.

Remarkably, GPs are most satisfied with colleagues and staff. This finding suggests strengthening team-based work in primary care; e.g. by improving work conditions within the practice team as well as by cooperating with other physicians outside one’s own practice, such as specialists or therapists. We were surprised to find an association of illegitimate tasks with life satisfaction. One explanation may be that illegitimate tasks are related to role identification and justification [28]. Possibly, the identification with one’s job is so strong among GPs that it results in a major overlap between life and job satisfaction. The association between illegitimate tasks and job satisfaction is in line with previous studies [17, 30]. Administrative tasks are risk factors for reduced job satisfaction [27, 30]. All in all, we can assume that an intervention to enhance life and job satisfaction should include elements that reduce illegitimate tasks.

Concerning the job demands-resources theory and mechanisms underlying the development of burnout symptoms, our findings suggest that illegitimate tasks as stressors are a significant contributor to develop burnout symptoms [23]. Work-SoC and recovery experience can be referred to as personal resources that may have a positive impact on stressors leading to burnout.

To reduce personal, work-related and patient-related burnout, our findings suggest that targeted interventions should focus on strengthening Work-SoC, recovery experience outside working hours and illegitimate tasks. In terms of personal burnout interventions should focus on gender issues (e.g. work-family interactions) and employed physicians (e.g. leadership). In terms of patient-related burnout, Balint groups fostering the exchange with colleagues may help enhance the relationship with patients [91, 92]. A benefit of enhancing health-promoting strategies among GPs is that those who learn about self-care are more likely to transfer their knowledge and strategies to their patients, and to include health promotion strategies in their patient interactions and consultations.

As regards unnecessary tasks, we need to question the existing workflow in order to find solutions to optimize processes and prevent or avoid those tasks. The large number of unreasonable tasks identified in our study indicates a need to reorganize tasks and responsibilities in general medical practice [33]. Worksharing between GPs and their staff and perhaps even colleagues, efficient task management, as well as an allocation of tasks according to profession, role and qualification may result in healthy and motivated health care professionals. Multi-professional and team-based working formats in primary care would be reasonable [93].

In research on burnout among physicians and other health care professionals, a large variety of instruments are applied to measure burnout [2]. The lack of a commonly accepted definition and instruments results in less comparable data. However, the CBI showed a good internal consistency and, unlike the MBI, allows us to draw more specific conclusions regarding personal, work-related and patient-related exhaustion. Therefore, this study encourages the use of other instruments than the MBI to quantify burnout symptoms. Our findings suggest to reconsider the definition of burnout that was published by the World Health Organization (WHO) in May 2019 and focuses solely on “chronic workplace stress that has not been successfully managed” [94].

### Limitations

We are aware that our research has limitations. First of all, due to the cross-sectional study design, we cannot draw conclusions on causal relationships. Secondly, a selection bias in our sample may be of concern. It is possible that particularly GPs with higher levels of satisfaction and happiness responded to a higher degree, or that less satisfied, unhappier and more highly stressed physicians wanted to share their perceptions with others. Moreover, GPs who have little time or who are stressed may actually have participated less frequently. However, a comparison of our findings with earlier research and existing studies still indicates reliable data [45, 51]. Our study population is mostly representative of the total population of German GPs in terms of sociodemographic and practice-related aspects [13]. However, our study participants more frequently work in group practices compared to the overall population of GPs. We tried to address a selection bias by offering an online and a paper-pencil survey. Taken together, we regard our sample rather balanced, i.e., less biased. Thirdly, the survey as a whole is based on self-reports (common method variance). Therefore, the participants may have responded in a socially desirable way. The fourth limitation relates to strong correlations between some predictors we included in the multiple linear regression. However, the assessment of multicollinearity [66] showed VIF values below 10 (personal burnout: average VIF = 1.158; work-related burnout: average VIF = 1.163; patient-related burnout: average VIF = 1.162). Fifthly, although our sample consisted of GPs working in Germany, we believe that our findings are relevant to similar occupational situations elsewhere, in other areas. Yet, specific health system-related aspects may account for differences, especially due to cultural differences in illegitimate tasks [28], which should be considered for further studies. Finally, as our study protocol, which was approved by the ethical review committee of Witten/Herdecke University (No. 134/2017), does not provide for sharing de-identified participant data outside the research team, we are not able to share data upon reasonable request. We will consider data sharing in future research. However, we will disseminate the results to study participants.

### Unanswered questions

As we measured satisfaction in two different ways–life satisfaction was conceptualized as general well-being, while job satisfaction was defined as an overall evaluation of one’s job–our research raises the question as to whether we need to develop a happiness measurement for work (specific happiness). The model fit indicates that happiness and life satisfaction are explained only to a small extent by the concepts of Work-SoC, recovery experience and illegitimate tasks. Future studies should examine other factors. There is an urgent need to identify the type of tasks that GPs consider unnecessary and unreasonable [33]. There is also a need for further research on which elements promote Work-SoC and recovery experience in the context of general medical practice. Our results suggest initiating a longitudinal study that allows monitoring changes in the future. This is relevant in the light of trends such as working in group practices or as employed physicians [16]. Following the analyses presented, further studies should assess the moderating influence of health-related resources on the correlation of stressors and mental health outcomes in terms of the job demands-resources theory [23, 39]. The next step should be to develop and evaluate targeted interventions for GPs to enhance life satisfaction, happiness and job satisfaction and to reduce burnout by applying our newly acquired knowledge of Work-SoC and recovery experience among GPs.

## Conclusion

Comparing our findings with those of other research studies allows us to conclude that German GPs are happier and more satisfied than other physician specialties or occupational groups in health care. Furthermore, we can conclude that burnout is not as severe among German GPs compared to physicians in general. Work-SoC, recovery experience and illegitimate tasks are associated with psychological well-being among GPs.

## Supporting information

S1 Table Linear model of predictors of life satisfaction, happiness and job satisfaction among GPs after multiple imputation. Bold = statistically significant at p< 0.05. b = unstandardized beta coefficient, SE = standard error of the unstandardized beta coefficient, β = standardized beta coefficient, p = p-value, CI = confidence interval, LL = lower level, UL = upper level. ^a^ 0 = Female, 1 = Male; ^b^ 0 = Single practice, 1 = Group practice; ^c^ 0 = Single practice, 1 = Medical care center; ^d^ 0 = Self-employed, 1 = Employed. Life satisfaction: Adjusted R^2^ = 0.48 (F = 23.530, p< 0.001). Happiness: Adjusted R^2^ = 0.206 (F = 18.798, p< 0.001). Job satisfaction: Adjusted R^2^ = 0.520 (F = 75.233, p< 0.001).
(DOCX)

S2 Table Linear models of predictors of burnout among GPs after multiple imputation. Bold = statistically significant at p< 0.05. b = unstandardized beta coefficient, SE = standard error of the unstandardized beta coefficient, β = standardized beta coefficient, p = p-value, CI = confidence interval, LL = lower level, UL = upper level. ^a^ 0 = Female, 1 = Male; ^b^ 0 = Single practice, 1 = Group practice; ^c^ 0 = Single practice, 1 = Medical care center; ^d^ 0 = Self-employed, 1 = Employed. Personal burnout: Adjusted R^2^ = 0.477 (F = 63.404, p< 0.001). Work-related burnout: Adjusted R^2^ = 0.566 (F = 90.320, p< 0.001). Patient-related burnout: Adjusted R^2^ = 0.310 (F = 31.794, p< 0.001).
(DOCX)

S3 Table Descriptive statistics of scales. M = Mean, SD = Standard deviation.
(DOCX)

S4 Table Aspects of job satisfaction among GPs. M = Mean, SD = Standard deviation.
(DOCX)
