# Supplementary material for: Burnout, satisfaction and happiness among German general practitioners (GPs): A cross-sectional survey on health resources and stressors
Source: PLoS One. 2021 Jun 18;16(6):e0253447. doi: 10.1371/journal.pone.0253447 (PMC8213182; doi:10.1371/journal.pone.0253447)
Supplement: S1 Table — Bold = statistically significant at p< 0.05. b = unstandardized beta coefficient, SE = standard error of the unstandardized beta coefficient, β = standardized beta coefficient, p = p-value, CI = confidence interval, LL = lower level, UL = upper level. a 0 = Female, 1 = Male; b 0 = Single practice, 1 = Group practice; c 0 = Single practice, 1 = Medical care center; d 0 = Self-employed, 1 = Employed. Life satisfaction: Adjusted R2 = 0.48 (F = 23.530, p< 0.001). Happiness: Adjusted R2 = 0.206 (F = 18.798, p< 0.001). Job satisfaction: Adjusted R2 = 0.520 (F = 75.233, p< 0.001). (DOCX) [file pone.0253447.s001.docx]

|  | Life satisfaction | | | | | | Happiness | | | | | | Job satisfaction | | | | | |
| --- | --- | --- | --- | --- | --- | --- | --- | --- | --- | --- | --- | --- | --- | --- | --- | --- | --- | --- |
| Effect | b | *SE* | 95% CI | | β | *p* | b | *SE* | 95% CI | | β | *p* | b | *SE* | 95% CI | | β | *p* |
|  |  |  | *LL* | *UL* |  |  |  |  | *LL* | *UL* |  |  |  |  | *LL* | *UL* |  |  |
| Intercept | 2.421 | 0.853 | 0.749 | 4.092 |  | **0.005** | 1.971 | 0.545 | 0.903 | 3.040 |  | **<0.001** | 2.037 | 0.372 | 1.308 | 2.767 |  | **<0.001** |
| Illegitimate tasks | -0.238 | 0.106 | -0.446 | -0.030 | -0.102 | **0.025** | -0.061 | 0.068 | -0.194 | 0.072 | -0.042 | 0.369 | -0.229 | 0.046 | -0.320 | -0.139 | -0.181 | **<0.001** |
| Work-SoC | 0.574 | 0.090 | 0.398 | 0.751 | 0.304 | **<0.001** | 0.260 | 0.057 | 0.148 | 0.373 | 0.221 | **<0.001** | 0.550 | 0.039 | 0.474 | 0.626 | 0.537 | **<0.001** |
| Recovery experience | 0.700 | 0.115 | 0.475 | 0.925 | 0.249 | **<0.001** | 0.549 | 0.074 | 0.403 | 0.695 | 0.314 | **<0.001** | 0.228 | 0.051 | 0.128 | 0.327 | 0.150 | **<0.001** |
| Male (ref. female) | -0.157 | 0.145 | -0.441 | 0.127 | -0.043 | 0.279 | -0.115 | 0.092 | -0.296 | 0.066 | -0.051 | 0.212 | -0.114 | 0.063 | -0.237 | 0.010 | -0.058 | 0.071 |
| Age^a^ | -0.005 | 0.008 | -0.021 | 0.012 | -0.023 | 0.579 | 0.002 | 0.005 | -0.008 | 0.013 | 0.019 | 0.658 | 0.005 | 0.004 | -0.002 | 0.013 | 0.051 | 0.140 |
| Group practice^b^ | 0.255 | 0.141 | -0.021 | 0.531 | 0.070 | 0.070 | 0.179 | 0.090 | 0.003 | 0.354 | 0.079 | **0.046** | 0.187 | 0.062 | 0.065 | 0.309 | 0.095 | **0.003** |
| Medical care center^c^ | 0.173 | 0.357 | -0.526 | 0.872 | 0.020 | 0.628 | 0.395 | 0.227 | -0.050 | 0.841 | 0.072 | 0.082 | 0.115 | 0.155 | -0.190 | 0.419 | 0.024 | 0.460 |
| Employed^d^ | -0.333 | 0.235 | -0.794 | 0.128 | -0.062 | 0.156 | -0.061 | 0.150 | -0.355 | 0.233 | -0.018 | 0.685 | -0.057 | 0.105 | -0.263 | 0.149 | -0.020 | 0.585 |
