# Supplementary material for: Burnout, satisfaction and happiness among German general practitioners (GPs): A cross-sectional survey on health resources and stressors
Source: PLoS One. 2021 Jun 18;16(6):e0253447. doi: 10.1371/journal.pone.0253447 (PMC8213182; doi:10.1371/journal.pone.0253447)
Supplement: S2 Table — Bold = statistically significant at p< 0.05. b = unstandardized beta coefficient, SE = standard error of the unstandardized beta coefficient, β = standardized beta coefficient, p = p-value, CI = confidence interval, LL = lower level, UL = upper level. a 0 = Female, 1 = Male; b 0 = Single practice, 1 = Group practice; c 0 = Single practice, 1 = Medical care center; d 0 = Self-employed, 1 = Employed. Personal burnout: Adjusted R2 = 0.477 (F = 63.404, p< 0.001). Work-related burnout: Adjusted R2 = 0.566 (F = 90.320, p< 0.001). Patient-related burnout: Adjusted R2 = 0.310 (F = 31.794, p< 0.001). (DOCX) [file pone.0253447.s002.docx]

|  | Personal burnout (n= 548) | | | | | | Work-related burnout (n= 548) | | | | | | Patient-related burnout (n= 548) | | | | | |
| --- | --- | --- | --- | --- | --- | --- | --- | --- | --- | --- | --- | --- | --- | --- | --- | --- | --- | --- |
| Effect | b | *SE* | β | 95% CI | | *p* | b | *SE* | β | 95% CI | | *p* | b | *SE* | β | 95% CI | | *p* |
|  |  |  |  | *LL* | *UL* |  |  |  |  | *LL* | *UL* |  |  |  |  | *LL* | *UL* |  |
| Intercept | 106.475 | 7.835 |  | 91.119 | 121.831 | **<0.001** | 101.468 | 6.832 |  | 88.077 | 114.860 | **<0.001** | 58.721 | 8.201 |  | 42.647 | 74.795 | **<0.001** |
| Illegitimate tasks | 5.384 | 0.979 | 0.210 | 3.465 | 7.304 | **<0.001** | 6.263 | 0.857 | 0.254 | 4.583 | 7.944 | **<0.001** | 5.559 | 1.020 | 0.238 | 3.560 | 7.559 | **<0.001** |
| Work-SoC | -6.088 | 0.829 | -0.292 | -7.713 | -4.463 | **<0.001** | -8.075 | 0.733 | -0.404 | -9.511 | -6.639 | **<0.001** | -6.442 | 0.858 | -0.340 | -8.124 | -4.759 | **<0.001** |
| Recovery experience | -11.523 | 1.057 | -0.373 | -13.594 | -9.452 | **<0.001** | -9.220 | 0.915 | -0.311 | -11.015 | -7.426 | **<0.001** | -3.173 | 1.106 | -0.113 | -5.341 | -1.004 | **0.004** |
| Male^a^ | -4.252 | 1.325 | -0.106 | -6.849 | -1.655 | **0.001** | -0.183 | 1.171 | -0.005 | -2.478 | 2.112 | 0.876 | 1.995 | 1.390 | 0.055 | -0.730 | 4.719 | 0.151 |
| Age | -0.131 | 0.076 | -0.060 | -0.280 | 0.018 | 0.086 | -0.154 | 0.066 | -0.074 | -0.284 | -0.024 | **0.020** | -0.074 | 0.079 | -0.037 | -0.229 | 0.081 | 0.352 |
| Group practice^b^ | -3.282 | 1.286 | -0.082 | -5.803 | -0.761 | **0.011** | -3.625 | 1.133 | -0.094 | -5.845 | -1.404 | **0.001** | -3.755 | 1.352 | -0.103 | -6.404 | -1.106 | **0.005** |
| Medical care center^c^ | -1.093 | 3.275 | -0.011 | -7.513 | 5.327 | 0.739 | -1.101 | 2.885 | -0.012 | -6.755 | 4.553 | 0.703 | -3.964 | 3.420 | -0.045 | -10.667 | 2.739 | 0.246 |
| Employed^d^ | 6.846 | 2.170 | 0.115 | 2.592 | 11.100 | 0.002 | -0.510 | 1.888 | -0.009 | -4.209 | 3.190 | 0.787 | 2.287 | 2.255 | 0.042 | -2.133 | 6.707 | 0.311 |
