# Supplementary material for: Burnout, satisfaction and happiness among German general practitioners (GPs): A cross-sectional survey on health resources and stressors
Source: PLoS One. 2021 Jun 18;16(6):e0253447. doi: 10.1371/journal.pone.0253447 (PMC8213182; doi:10.1371/journal.pone.0253447)
Supplement: S3 Table — M = Mean, SD = Standard deviation. (DOCX) [file pone.0253447.s003.docx]

|  | **Item** | **n** | **M** | **SD** |
| --- | --- | --- | --- | --- |
| **Subjective Happiness Scale** | SHS_1 | 546 | 5.49 | 1.11 |
|  | SHS_2 | 547 | 5.40 | 1.25 |
|  | SHS_3 | 547 | 4.87 | 1.41 |
|  | SHS_4 | 547 | 4.78 | 2.23 |
| **Job satisfaction** | Job satisfaction_1 | 546 | 5.31 | 1.46 |
|  | Job satisfaction_2 | 547 | 5.27 | 1.46 |
|  | Job satisfaction_3 | 539 | 5.79 | 1.20 |
|  | Job satisfaction_4 | 547 | 5.37 | 1.35 |
|  | Job satisfaction_5 | 548 | 5.46 | 1.45 |
|  | Job satisfaction_6 | 548 | 4.93 | 1.72 |
|  | Job satisfaction_7 | 548 | 5.59 | 1.33 |
|  | Job satisfaction_8 | 546 | 4.27 | 1.91 |
|  | Job satisfaction_9 | 547 | 5.51 | 1.30 |
|  | Job satisfaction_10 | 545 | 5.60 | 1.18 |
| **Berne Illegitimate Task Scale** | Illegitimate Tasks_1 | 546 | 3.37 | 0.93 |
|  | Illegitimate Tasks_2 | 545 | 3.47 | 0.93 |
|  | Illegitimate Tasks_3 | 544 | 3.47 | 0.98 |
|  | Illegitimate Tasks_4 | 546 | 3.65 | 1.11 |
|  | Illegitimate Tasks_5 | 547 | 3.06 | 0.92 |
|  | Illegitimate Tasks_6 | 545 | 2.82 | 1.03 |
|  | Illegitimate Tasks_7 | 547 | 2.20 | 0.96 |
|  | Illegitimate Tasks_8 | 547 | 2.42 | 1.10 |
| **Work-SoC** | Work-SoC_1 | 546 | 5.66 | 1.54 |
|  | Work-SoC_2 | 546 | 6.16 | 1.12 |
|  | Work-SoC_3 | 547 | 5.55 | 1.32 |
|  | Work-SoC_4 | 546 | 4.75 | 1.53 |
|  | Work-SoC_5 | 547 | 5.90 | 1.07 |
|  | Work-SoC_6 | 547 | 5.08 | 1.50 |
|  | Work-SoC_7 | 546 | 4.58 | 1.62 |
|  | Work-SoC_8 | 546 | 5.69 | 1.43 |
|  | Work-SoC_9 | 547 | 4.16 | 1.56 |
| **Recovery Experience Questionnaire** | REQ_1 | 548 | 3.36 | 0.98 |
|  | REQ_2 | 547 | 2.93 | 1.12 |
|  | REQ_3 | 547 | 3.60 | 0.97 |
|  | REQ_4 | 547 | 3.56 | 0.95 |
|  | REQ_5 | 545 | 3.22 | 1.01 |
|  | REQ_6 | 548 | 3.52 | 0.94 |
|  | REQ_7 | 548 | 3.22 | 1.01 |
|  | REQ_8 | 546 | 3.05 | 1.05 |
|  | REQ_9 | 546 | 3.24 | 0.91 |
|  | REQ_10 | 547 | 3.02 | 1.06 |
|  | REQ_11 | 547 | 3.02 | 1.00 |
|  | REQ_12 | 546 | 3.27 | 0.95 |
|  | REQ_13 | 545 | 3.62 | 1.02 |
|  | REQ_14 | 546 | 3.54 | 1.01 |
|  | REQ_15 | 546 | 3.56 | 0.99 |
|  | REQ_16 | 547 | 3.50 | 0.97 |
| **Personal burnout** | CBI_personal_1 | 547 | 61.61 | 23.64 |
|  | CBI_personal_2 | 547 | 51.51 | 24.65 |
|  | CBI_personal_3 | 548 | 49.64 | 26.53 |
|  | CBI_personal_4 | 546 | 24.31 | 26.29 |
|  | CBI_personal_5 | 547 | 42.55 | 27.12 |
|  | CBI_personal_6 | 546 | 25.46 | 22.76 |
| **Work-related burnout** | CBI_work_1 | 547 | 58.09 | 26.51 |
|  | CBI_work_2 | 546 | 29.03 | 25.79 |
|  | CBI_work_3 | 547 | 22.76 | 22.10 |
|  | CBI_work_4 | 545 | 40.09 | 25.69 |
|  | CBI_work_5 | 546 | 58.97 | 24.36 |
|  | CBI_work_6 | 546 | 25.05 | 23.04 |
|  | CBI_work_7 | 546 | 26.01 | 26.77 |
| **Patient-related burnout** | CBI_patient_1 | 547 | 19.84 | 21.87 |
|  | CBI_patient_2 | 546 | 34.66 | 28.68 |
|  | CBI_patient_3 | 544 | 19.49 | 20.90 |
|  | CBI_patient_4 | 546 | 39.01 | 27.31 |
|  | CBI_patient_5 | 545 | 17.84 | 21.42 |
|  | CBI_patient_6 | 546 | 29.12 | 27.78 |
