# Supplementary material for: Burnout, satisfaction and happiness among German general practitioners (GPs): A cross-sectional survey on health resources and stressors
Source: PLoS One. 2021 Jun 18;16(6):e0253447. doi: 10.1371/journal.pone.0253447 (PMC8213182; doi:10.1371/journal.pone.0253447)
Supplement: S4 Table — M = Mean, SD = Standard deviation. (DOCX) [file pone.0253447.s004.docx]

| Variables | *M* | *SD* | *Missing data* |
| --- | --- | --- | --- |
| Physical work conditions | 5.31 | 1.46 | 2 |
| Freedom of working method | 5.27 | 1.46 | 1 |
| Colleagues and staff | 5.79 | 1.20 | 9 |
| Recognition for work | 5.37 | 1.35 | 1 |
| Level of responsibility | 5.46 | 1.45 | 0 |
| Income | 4.93 | 1.72 | 0 |
| Opportunity to use abilities | 5.59 | 1.33 | 0 |
| Hours of work | 4.27 | 1.91 | 2 |
| Variety in job | 5.51 | 1.30 | 1 |
| Overall job satisfaction | 5.60 | 1.18 | 3 |
